# Successful Treatment of Pediatric Holo-Spinal Epidural Abscess With Percutaneous Drainage

**DOI:** 10.7759/cureus.24735

**Published:** 2022-05-04

**Authors:** Adam A Ammar, Mousa K Hamad, Malik S Obeidallah, Andrew J Kobets, Seon-Kyu Lee, Ira R Abbott

**Affiliations:** 1 Department of Neurological Surgery, Montefiore Medical Center, Moses Campus, Bronx, USA; 2 Department of Neurointerventional Radiology, Montefiore Medical Center, Moses Campus, Bronx, USA

**Keywords:** conservative management, percutaneous drainage, mrsa, treatment of spinal epidural abscess, holocord

## Abstract

Spinal epidural abscess (SEA) is a rare and potentially devastating neurologic disease that is commonly treated with neurosurgical decompression and evacuation. We describe the case of an 11-month-old immunocompetent infant who presented with a large multiloculated methicillin-resistant *Staphylococcus aureus* abscess in the left lung apex with likely mediastinal involvement, extending into the epidural space from C7 down to L2 causing cord compression which was successfully treated with percutaneous placement of an epidural drainage catheter and antibiotic therapy. Although there are rare reports of percutaneous drainage of SEAs, to our knowledge, there are no reports of successful use of percutaneous indwelling catheters resulting in the complete resolution of an SEA. Holo-spinal epidural abscess in an infant is an extremely rare disease with limited literature available regarding the best practice for its treatment. Multiple considerations must be taken into account when weighing the different treatment options ranging from surgical decompression to conservative management with antibiotic therapy. We present a unique case of successful treatment with percutaneous epidural drain placement. This provides a reasonable alternative for management in children for whom surgical decompression carries multiple risks for complications both acutely and delayed.

## Introduction

Spinal epidural abscess (SEA) is a rare but potentially devastating neurologic disease that is commonly treated with neurosurgical decompression and evacuation. The incidence ranges from 0.2 to 1.2 patients per 10,000 hospital admissions, but only a few percent of these cases occur in children, with holocord epidural abscess even rarer with fewer than a dozen cases reported in the literature [1]. Early diagnosis and prompt treatment are critical, with an excellent prognosis if treated in a timely fashion [2]. However, this can be difficult in the pediatric population given the nonspecific signs and symptoms in infants leading to delays in diagnosis [3]. We describe the case of an 11-month-old immunocompetent infant who presented with a holocord posterior spinal epidural methicillin-resistant *Staphylococcus aureus* (MRSA) abscess and was successfully treated with percutaneous placement of an epidural drainage catheter and antibiotic therapy. Although there are rare reports of percutaneous drainage of SEAs, to our knowledge, there are no reports of successful use of percutaneous indwelling catheters for complete drainage of an SEA [4].

## Case presentation

History of present illness

An 11-month-old ex-full-term baby boy with no medical history presented to our institution with three days of fever and decreased oral intake. He had had a viral upper respiratory infection 10 days prior to presentation. He was fully vaccinated, including the flu vaccine for the year, and had no sick contacts, including no one in the household with symptoms concerning for coronavirus disease 2019 (COVID-19).

Examination

The baby was noted to be dehydrated with tachypnea unresponsive to fluid resuscitation, requiring a high-flow nasal cannula. Neurologic examination was unremarkable.

Workup and imaging

**Figure 1:**
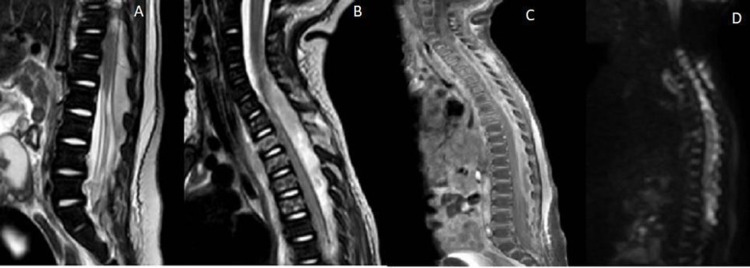
T2 Sagittal MRI of the thoracolumbar (A) and cervicothoracic (B) regions demonstrate posterior epidural collection causing significant cord compression. Post-contrast (C) and DWI (D) imaging confirm the diagnosis of spinal epidural abscess. MRI: magnetic resonance imaging; DWI: diffusion-weighted imaging

Chest X-ray demonstrated left upper lobe pneumonia, and blood cultures grew MRSA. Lumbar puncture confirmed meningitis as well. COVID-19 was negative on two separate tests. Given persistent fevers and meningitis one week after presentation, imaging of the entire neuroaxis was obtained, demonstrating a large multiloculated abscess in the left lung apex with likely mediastinal involvement, extending into the epidural space from C7 down to L2 causing cord compression (Figure 1). Post-contrast imaging demonstrated heterogeneous enhancement of the collection, and diffusion-weighted imaging showed restricted diffusion, confirming the suspicion of epidural abscess.

Intervention

**Figure 2:**
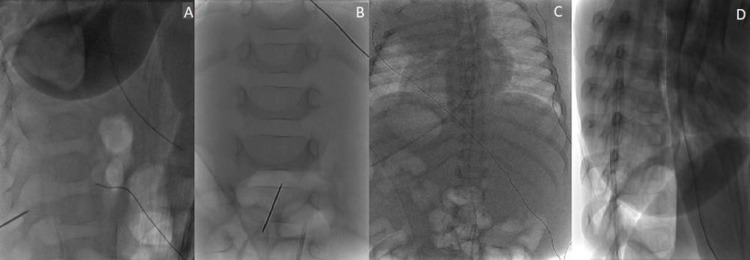
Lateral (A) and posteroanterior (B) radiographs demonstrating needle placement at the L2-3 interspace. Confirmatory posteroanterior (C) and lateral (D) radiographs demonstrate the epidural catheter extending up to approximately the T7 level.

**Figure 3:**
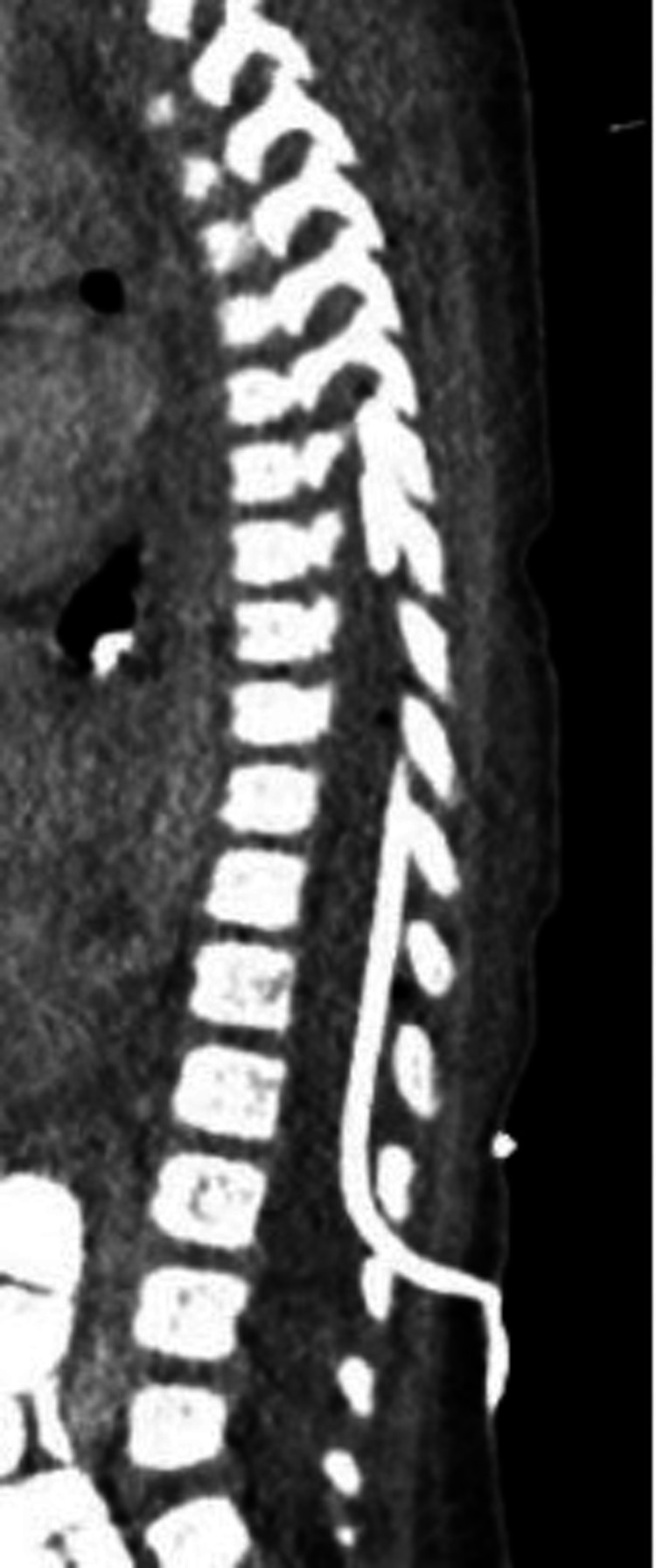
Postoperative CT demonstrating catheter in the epidural space. CT: computed tomography

Given the patient’s intact neurologic examination and the extent of the epidural abscess, percutaneous drainage of the abscess was opted for rather than surgical decompression and evacuation. The patient was taken to the neurointerventional radiology suite and placed prone on the biplane neuroangiography table. An 18-gauge spinal needle was advanced to the L2-3 epidural space under fluoroscopic guidance and, subsequently, cone-beam computed tomography was obtained to confirm the needle position (Figure 2). A 6-French dilator was then slowly introduced to dilate the track, and a 6-French pig-tail drainage tube was slowly introduced mainly on the left side of the thoracolumbar epidural space and advanced to around the T7 area (Figure 3). Approximately 3 mL of pus was drained immediately, and the drainage catheter was attached to a buretrol system which was maintained at 10 cm below the level of the abdomen. The drain was left in place for one week with the slow removal of 1-2 cm per day starting on postoperative day three until the distal pig-tail was removed under fluoroscopic guidance on postoperative day seven.

Outcome

**Figure 4:**
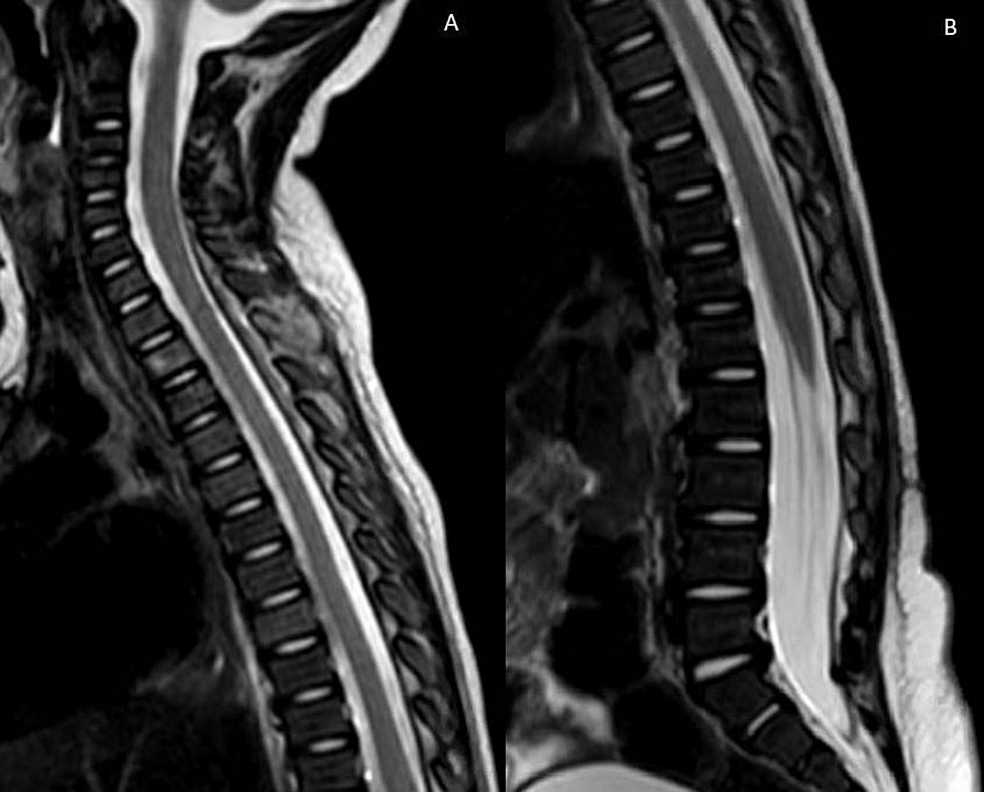
Three-week postoperative T2 MRI of the cervicothoracic (A) and thoracolumbar (B) regions demonstrate near-complete resolution of the spinal epidural abscess. MRI: magnetic resonance imaging

The patient remained neurologically intact during his hospitalization. Repeat magnetic resonance imaging three weeks after drain placement demonstrated near-complete resolution of the epidural abscess (Figure 4), and the patient was able to be discharged on long-term antibiotic therapy one month after treatment. An extensive immunology workup demonstrated no findings concerning for an underlying immunodeficiency.

## Discussion

Epidemiology

The incidence of SEA is low ranging from 0.2 to 1.2 patients per 10,000 hospital admissions and is more common in adults than in children with a peak between 50 and 70 years old [3,5]. The mean age of SEA in children is about eight years [6] and is usually related to previous lumbar puncture, epidural anesthesia, or spinal surgery [7]. In adults, it usually occurs in those with predisposing conditions such as diabetes mellitus, chronic renal failure, cancer, immunodeficiency, alcoholism, and intravenous drug abuse, with diabetes mellitus being the most common risk factor. Pediatric risk factors include sickle cell anemia, leukemia, long-term use of steroids, and other causes of immunodeficiency [4,8]. The usual location for SEA is the mid-thoracic to the lumbar spine due to the narrowing of the spinal cord at these levels leading to a larger epidural space; the epidural venous plexus is the presumed route of infection. Most SEAs are dorsal, with ventral abscesses being rare because of the adherence of the posterior longitudinal ligament to the dura mater ventrally, leaving a small potential space [5].

Etiology and pathophysiology

The etiology of SEA is bacterial spread to the epidural space through contiguous spread (primary SEA) or hematogenous dissemination (secondary SEA). The most frequent cause in children is hematogenous spread [2,8,9]. Extensive or holocord SEA is more commonly seen in children due to more fat and septation in the epidural space compared to adults. Children also have more vascularity around the vertebral body predisposing them to the hematogenous spread of bacteria [4,10]. The most common causative agent is *Staphylococcus aureus*, accounting for 50-90% of cases, followed by *Streptococcus* (8-17%), and Gram-negative bacteria (10-17%) [11].

Diagnosis and workup

Clinical presentation of SEA is variable with the classic triad of fever, back pain, and rarely, neurological deficit rarely (10-15% of cases) the initial form of presentation. The diagnosis is even more challenging in children. The following four clinical stages have been described in the progression of the disease: (1) spinal pain, fever, and local tenderness are the presenting symptoms; (2) radicular pain, nuchal rigidity, and changes in the reflexes then appear; (3) sensory and motor abnormalities arise, including bowel and bladder dysfunction, as the disease goes untreated; and (4) paralysis then occurs as neurologic deficits become permanent. Alterations are reversible up to stage four, and paralysis lasting longer than 24-36 hours is unlikely to improve [5].

Laboratory tests include markers of inflammation such as leukocyte count (white blood cell count), erythrocyte sedimentation rate (ESR), and C-reactive protein (CRP), and studies in adult and pediatric patient populations have noted increases in these inflammatory markers in patients with SEA. Acute-phase reactants such as ESR and CRP are stronger indicators than leukocyte count [2,4]. Workup for an underlying predisposition should also be pursued, particularly for any immunodeficiency. For imaging, once SEA is suspected, a timely spinal MRI with and without contrast is an excellent tool to confirm the diagnosis [2]. Lastly, confirmatory microbiologic diagnosis is essential and can be achieved by blood culture or direct biopsy of the abscess.

Treatment and management

There is no consensus on the optimal treatment of SEA as it is confounded by clinical heterogeneity [4,12]. Conflicting systematic reviews have concluded no significant difference in outcomes between operative and nonoperative management in patients presenting without neurologic deficit or spinal instability [13], and improved outcomes with urgent surgical decompression, as 49% of patients managed nonoperatively decompensated [14]. Based on the available adult literature, four scenarios have been proposed in which nonsurgical management may be considered: (1) patients without neurologic deficit, (2) extensive or holocord SEA that would require a multilevel laminectomy, (3) greater than 48 hours of paraplegia as the chance of neurologic recovery diminishes greatly, and (4) extensive comorbid conditions that increase the risk of surgery [4,15].

Management of SEA in the pediatric population as well as holocord SEA is even more complicated, and there are no official recommendations or guidelines regarding their management as these entities are rare and data are limited [16,17]. The extensive laminectomy advised for the treatment of SEA in adults is undesirable in children as widespread decompression potentially predisposes patients to complications such as instability, postoperative low-back pain, cardiac and respiratory difficulties, and late kyphotic deformity [4]. Consideration of these complications is particularly important in the pediatric population given their skeletal immaturity and longer life expectancy [5]. Given this concern, there have been multiple reports of skip, limited and apical laminectomies with or without catheter irrigation, and of conservative management with antibiotic therapy alone without drainage [5,18-27].

## Conclusions

Holo-spinal epidural abscess in an infant is an extremely rare disease with limited literature available regarding the best practice for its treatment. Multiple considerations must be taken into account when weighing the different treatment options ranging from surgical decompression to conservative management with antibiotic therapy. We present a unique case of successful treatment with percutaneous epidural drain placement. This provides a reasonable alternative for management in children for whom surgical decompression carries multiple risks for complications both acutely and delayed.

## References

[1] Jacobsen FS, Sullivan B (1994). Spinal epidural abscesses in children. Orthopedics.

[2] Ghosh PS, Loddenkemper T, Blanco MB, Marks M, Sabella C, Ghosh D (2009). Holocord spinal epidural abscess. J Child Neurol.

[3] Fischer EG, Greene CS Jr, Winston KR (1981). Spinal epidural abscess in children. Neurosurgery.

[4] Walter RS, King JC Jr, Manley J, Rigamonti D (1991). Spinal epidural abscess in infancy: successful percutaneous drainage in a nine-month-old and review of the literature. Pediatr Infect Dis J.

[5] Spennato P, Renedo D, Cascone D, Mirone G, Imperato A, Di Martino G, Cinalli G (2020). Spinal epidural abscess in children: a case-based review. Childs Nerv Syst.

[6] Mohanty CB, Fieggen G, Deopujari CE (2018). Pediatric spinal infections-a review of non-tuberculous infections. Childs Nerv Syst.

[7] Tang K, Xenos C, Sgouros S (2001). Spontaneous spinal epidural abscess in a neonate. With a review of the literature. Childs Nerv Syst.

[8] Fotaki A, Anatoliotaki M, Tritou I, Tzagaraki A, Kampitaki M, Vlachaki G (2019). Review and case report demonstrate that spontaneous spinal epidural abscesses are rare but dangerous in childhood. Acta Paediatr.

[9] Hawkins M, Bolton M (2013). Pediatric spinal epidural abscess: a 9-year institutional review and review of the literature. Pediatrics.

[10] Auletta JJ, John CC (2001). Spinal epidural abscesses in children: a 15-year experience and review of the literature. Clin Infect Dis.

[11] Vergori A, Cerase A, Migliorini L (2015). Pediatric spinal epidural abscess in an immunocompetent host without risk factors: case report and review of the literature. IDCases.

[12] Kurudza E, Stadler JA 3rd (2019). Pediatric holocord epidural abscess treated with apical laminotomies with catheter-directed irrigation and drainage. Cureus.

[13] Arko L 4th, Quach E, Nguyen V, Chang D, Sukul V, Kim BS (2014). Medical and surgical management of spinal epidural abscess: a systematic review. Neurosurg Focus.

[14] Curry WT Jr, Hoh BL, Amin-Hanjani S, Eskandar EN (2005). Spinal epidural abscess: clinical presentation, management, and outcome. Surg Neurol.

[15] Leys D, Lesoin F, Viaud C, Pasquier F, Rousseaux M, Jomin M, Petit H (1985). Decreased morbidity from acute bacterial spinal epidural abscesses using computed tomography and nonsurgical treatment in selected patients. Ann Neurol.

[16] Pradilla G, Nagahama Y, Spivak AM, Bydon A, Rigamonti D (2010). Spinal epidural abscess: current diagnosis and management. Curr Infect Dis Rep.

[17] Houston R, Gagliardo C, Vassallo S, Wynne PJ, Mazzola CA (2019). Spinal epidural abscess in children: case report and review of the literature. World Neurosurg.

[18] Urrutia J, Rojas C (2007). Extensive epidural abscess with surgical treatment and long term follow up. Spine J.

[19] Xiang H, Ma X, Shen N, Yue B, Zhang G, Chen B (2016). Holocord spinal epidural abscess: case report and literature review. Orthop Traumatol Surg Res.

[20] Abd-El-Barr MM, Bi WL, Bahluyen B, Rodriguez ST, Groff MW, Chi JH (2015). Extensive spinal epidural abscess treated with "apical laminectomies" and irrigation of the epidural space: report of 2 cases. J Neurosurg Spine.

[21] Ahuja K, Das L, Jain A, Meena PK, Arora SS, Kandwal P (2019). Spinal holocord epidural abscess evacuated with double thoracic interval laminectomy: a rare case report with literature review. Spinal Cord Ser Cases.

[22] Lam KS, Pande KC, Mehdian H (1997). Surgical decompression: a life-saving procedure for an extensive spinal epidural abscess. Eur Spine J.

[23] Leonard J, Kaufman B (2001). Treatment of a holocord epidural abscess. Case illustration. J Neurosurg.

[24] Hwang R, Yung BH, Sedney C, Miele VJ (2015). Treatment of holocord spinal epidural abscess via alternating side unilateral approach for bilateral laminectomy. W V Med J.

[25] Supreeth S, Al Ghafri K (2019). Ventral holocord spinal epidural abscess managed surgically in a critical setting. Surg Neurol Int.

[26] O'Brien C, Lenehan B, Street J (2011). Non-operative management of an extensive anteriorly located epidural abscess. J Clin Neurosci.

[27] Pathak A, Singh P, Gehlot P, Dhaneria M (2013). Spinal epidural abscess treated with antibiotics alone. BMJ Case Rep.

